# Genome-Resolved Metagenomics of a Photosynthetic Bioreactor Performing Biological Nutrient Removal

**DOI:** 10.1128/MRA.00244-21

**Published:** 2021-05-06

**Authors:** Elizabeth A. McDaniel, Renske Wever, Ben O. Oyserman, Daniel R. Noguera, Katherine D. McMahon

**Affiliations:** a Department of Bacteriology, University of Wisconsin—Madison, Madison, Wisconsin, USA; b Department of Civil and Environmental Engineering, University of Wisconsin—Madison, Madison, Wisconsin, USA; c Department of Microbial Ecology, Netherlands Institute of Ecological Research, Wageningen, The Netherlands; d Bioinformatics Group, Wageningen University and Research, Wageningen, The Netherlands; e DOE Great Lakes Bioenergy Research Center, Madison, Wisconsin, USA; University of Southern California

## Abstract

Enhanced biological phosphorus removal (EBPR) is an economically and environmentally significant wastewater treatment process for removing excess phosphorus by harnessing the metabolic physiologies of enriched microbial communities. We present a genome-resolved metagenomic data set consisting of 86 metagenome-assembled genome sequences from a photosynthetically operated lab-scale bioreactor simulating EBPR.

## ANNOUNCEMENT

A photobioreactor performing light-supported enhanced biological phosphorus removal (EBPR) was seeded from two lab-scale wastewater treatment systems, a photosynthetic nitrifying enrichment culture and an activated sludge EBPR bioreactor designed to enrich for the polyphosphate-accumulating organism “*Candidatus* Accumulibacter phosphatis” (1, 2). The photobioreactor was designed to achieve phosphorus removal and denitrification without the addition of mechanical aeration by providing light (2). Three samples were collected during cycles 87, 103, and 129. DNA was extracted as described (2), and 100 ng of DNA was sheared to 300 bp using the Covaris LE220 ultrasonicator and size selected with solid-phase reversible immobilization (SPRI) beads (Beckman Coulter). The fragments were ligated with end repair, A-tailing, and Illumina-compatible adapters (IDT, Inc.) using the KAPA-Illumina library preparation kit (Kapa Biosystems). The libraries were quantified using the Kapa Biosystems next-generation sequencing library quantitative PCR (qPCR) kit and run on a Roche LightCycler 480 real-time PCR instrument. The quantified libraries were prepared using the v4 TruSeq paired-end cluster kit and the Illumina cBot instrument to create a clustered flow cell for sequencing. Shotgun metagenomic sequencing was performed on the Illumina HiSeq 2500 platform with the TruSeq sequencing-by-synthesis (SBS) sequencing kit, followed by 2 × 150-bp indexing. All metagenomic libraries consist of approximately 50 million 150-bp paired-end reads (table of metagenome information available at https://doi.org/10.6084/m9.figshare.14164307).

**FIG 1:**
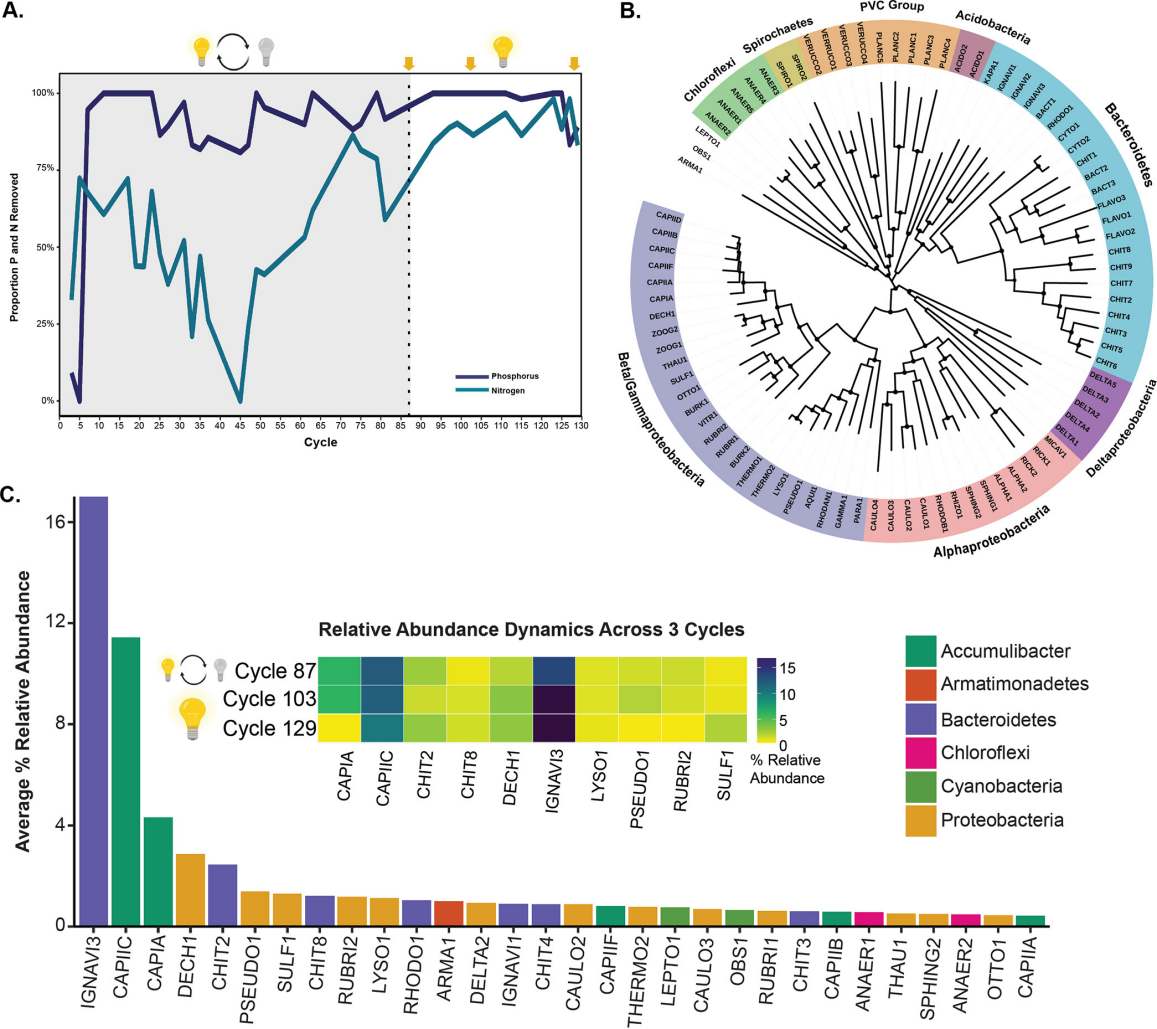
Overview of reactor performance and assembled MAGs. (A) Proportions of nitrogen (blue) and phosphorus (purple) removed during reactor operation. Cycle length refers to the hydraulic residence time of 0.625 days (2). The reactor was operated in two phases—in the first phase (gray), light was cycled on and off, and in the second phase (white), light was provided continuously. The arrows denote the time points at which metagenomic sequencing was performed. (B) Phylogenetic tree of all 86 assembled MAGs constructed from a concatenated alignment of 120 single-copy marker genes from GTDB-tk v0.3.2 (9) that were identified and aligned using HMMER v3.2.1 (11). The tree was constructed using RAxML v8.2.12 with 100 rapid bootstraps (12) and viewed using iTOL v5.7 (13). (C) Relative abundance and population dynamics of abundant species represented by assembled MAGs. The bar graph represents the average relative abundance of the top 30 most abundant species across all three samples. Relative abundance calculations were made using coverM v0.4.0 with the relative_abundance method and averaged across all three samples. The inset heat map shows the population dynamics of the top 10 most abundant species, representing the relative abundance of that MAG in that sample.

Default parameters were used for all software unless otherwise specified. The raw metagenomic reads were quality filtered and trimmed using bbduk as part of the BBTools v38.07 suite (3). All three metagenomic samples were coassembled and individually assembled into contigs using SPAdes v3.9.0 with the metagenomic option (4). Metagenomic reads from each sample were mapped against all assemblies using BBMap as part of the BBTools v38.07 suite with a 95% sequence identity cutoff (3). The assembled contigs from each assembly were binned into population genomes using MetaBAT2 v2.12.1 using only contigs larger than 1,000 bp (5). The bins were dereplicated using dRep v2.4.2 (6) to obtain the highest-quality representative set of genomes. This resulted in a total of 86 nonredundant species-resolved genome sequences, described in Table 1 and summarized in Fig. 1. All genome sequences were checked for uniform differential coverage using uBin v0.9.14 (7). All genome statistics were calculated with CheckM v1.1.2 (8). Taxonomical classifications were made based on the Genome Taxonomy Database (GTDB) using GTDB-Tk v0.3.2 (9, 10). Relative abundance calculations were performed with coverM v0.4.0 using the relative_abundance calculation method. The methods and phylogenetic tree for assigning clades of six “*Ca.* Accumulibacter phosphatis” draft genome sequences based on the *ppk1* locus are available at https://doi.org/10.6084/m9.figshare.14164478.

**TABLE 1:** Genome accession numbers and statistics^*a*^

															Relative abundance on date (mo/day/yr)						
Isolate name	Assembly accession no.	Code	GTDB classification	Completeness (%)	Contamination (%)	Genome size (mbp)	No. of contigs	Avg contig size (bp)	% GC content	No. of rRNAs	No. of tRNAs	No. of 5S rRNAs	No. of 16S rRNAs	No. of 23S rRNAs	7/16/2015	7/24/2015	8/6/2015	Avg abundance	BioSample accession no.	GenBank accession no.	BioProject accession no.
UWPOB_ACIDO1	GCA_017304135.1	ACIDO1	d__Bacteria;p__Acidobacteriota;c__Acidobacteriae;o__Bryobacterales;f__Bryobacteraceae;g__;s__	98.4	2.9	6.49	365	17,791	64.5	3	48	1	1	1	0.04386	0.06137	0.15309	0.08611	SAMN18059832	JAFLBB000000000	PRJNA704939
UWPOB_ACIDO2	GCA_017303955.1	ACIDO2	d__Bacteria;p__Acidobacteriota;c__Blastocatellia;o__UBA7656;f__;g__;s__	100	2.1	6.15	46	133,664	34.5	0	83	0	0	0	0.01576	0.0143	0.30104	0.11037	SAMN18059833	JAFLBC000000000	PRJNA704939
UWPOB_ARMA1	GCA_017303935.1	ARMA1	d__Bacteria;p__Armatimonadota;c__Fimbriimonadia;o__Fimbriimonadales;f__Fimbriimonadaceae;g__UBA2391;s__	100	0	2.56	5	511,480	53.2	3	47	1	1	1	1.87686	0.86294	0.31483	1.01821	SAMN18059834	JAFLBD000000000	PRJNA704939
UWPOB_BACT1	GCA_017303995.1	BACT1	d__Bacteria;p__Bacteroidota;c__;o__;f__;g__;s__	100	0	4.31	137	31,460	41.5	2	40	2	0	0	0.12322	0.02724	0.08244	0.07763	SAMN18059835	JAFLBE000000000	PRJNA704939
UWPOB_BACT2	GCA_017303975.1	BACT2	d__Bacteria;p__Bacteroidota;c__Bacteroidia;o__AKYH767;f__2-12-FULL-35-15;g__;s__	95.7	1	3.47	75	46,223	35.2	3	32	1	1	1	0.12227	0.02794	0.02315	0.05779	SAMN18059837	JAFLBG000000000	PRJNA704939
UWPOB_BACT3	GCA_017303895.1	BACT3	d__Bacteria;p__Bacteroidota;c__Bacteroidia;o__AKYH767;f__2-12-FULL-35-15;g__;s__	99.1	0.7	3.61	35	103,001	37.3	3	33	1	1	1	0.10972	0.06722	0.02034	0.06576	SAMN18059836	JAFLBF000000000	PRJNA704939
UWPOB_CHIT1	GCA_017303905.1	CHIT1	d__Bacteria;p__Bacteroidota;c__Bacteroidia;o__AKYH767;f__b-17BO;g__;s__	100	0	3.95	7	564,856	36.8	4	33	2	1	1	0.08039	0.12427	0.26855	0.15774	SAMN18059838	JAFLBH000000000	PRJNA704939
UWPOB_CHIT2	GCA_017303835.1	CHIT2	d__Bacteria;p__Bacteroidota;c__Bacteroidia;o__Chitinophagales;f__Chitinophagaceae;g__;s__	99.5	0.3	3.26	2	1,631,117	42.5	3	36	1	1	1	2.73174	1.62266	3.05499	2.4698	SAMN18059839	JAFLBI000000000	PRJNA704939
UWPOB_CHIT3	GCA_017303415.1	CHIT3	d__Bacteria;p__Bacteroidota;c__Bacteroidia;o__Chitinophagales;f__Chitinophagaceae;g__;s__	100	0	3.72	3	1,239,140	46	1	37	0	1	0	0.64668	0.61427	0.58909	0.61668	SAMN18059840	JAFLBJ000000000	PRJNA704939
UWPOB_CHIT4	GCA_017303335.1	CHIT4	d__Bacteria;p__Bacteroidota;c__Bacteroidia;o__Chitinophagales;f__Chitinophagaceae;g__Ferruginibacter;s__	99	0.3	4.01	7	572,270	49.5	3	42	1	1	1	0.64536	0.73815	1.22698	0.87016	SAMN18059841	JAFLBK000000000	PRJNA704939
UWPOB_CHIT5	GCA_017303455.1	CHIT5	d__Bacteria;p__Bacteroidota;c__Bacteroidia;o__Chitinophagales;f__Chitinophagaceae;g__JJ008;s__	95.8	0	4.55	57	79,888	43	0	40	0	0	0	0.0768	0.06206	0.05275	0.06387	SAMN18059843	JAFLBM000000000	PRJNA704939
UWPOB_CHIT6	GCA_017303855.1	CHIT6	d__Bacteria;p__Bacteroidota;c__Bacteroidia;o__Chitinophagales;f__Chitinophagaceae;g__JJ008;s__	99.5	0.7	4.62	31	149,027	46.1	1	37	1	0	0	0.23357	0.2022	0.15554	0.1971	SAMN18059842	JAFLBL000000000	PRJNA704939
UWPOB_CHIT7	GCA_017303395.1	CHIT7	d__Bacteria;p__Bacteroidota;c__Bacteroidia;o__Chitinophagales;f__Chitinophagaceae;g__Taibaiella_B;s__	100	6.3	3.76	45	83,566	41.9	0	33	0	0	0	0.12824	0.08724	0.02842	0.0813	SAMN18059844	JAFLBN000000000	PRJNA704939
UWPOB_CHIT8	GCA_017303475.1	CHIT8	d__Bacteria;p__Bacteroidota;c__Bacteroidia;o__Chitinophagales;f__Saprospiraceae;g__;s__	98.5	0.5	6.04	53	114,008	47.9	3	40	1	1	1	0.83528	1.32521	1.47064	1.21038	SAMN18059845	JAFLBO000000000	PRJNA704939
UWPOB_CHIT9	GCA_017303875.1	CHIT9	d__Bacteria;p__Bacteroidota;c__Bacteroidia;o__Chitinophagales;f__Saprospiraceae;g__;s__	99	0.5	5.78	64	90,342	50.4	0	43	0	0	0	0.12036	0.15272	0.42621	0.2331	SAMN18059846	JAFLBP000000000	PRJNA704939
UWPOB_CYTO1	GCA_017303805.1	CYTO1	d__Bacteria;p__Bacteroidota;c__Bacteroidia;o__Cytophagales;f__Cyclobacteriaceae;g__ELB16-189;s__	98.5	0.3	4.29	13	329,632	45.1	1	35	1	0	0	0.03105	0.06252	0.27364	0.1224	SAMN18059847	JAFLBQ000000000	PRJNA704939
UWPOB_CYTO2	GCA_017303795.1	CYTO2	d__Bacteria;p__Bacteroidota;c__Bacteroidia;o__Cytophagales;f__Cyclobacteriaceae;g__ELB16-189;s__	100	0	4.87	27	180,295	42.2	2	34	1	0	1	0.06209	0.0432	0.20485	0.10338	SAMN18059848	JAFLBR000000000	PRJNA704939
UWPOB_FLAVO1	GCA_017302935.1	FLAVO1	d__Bacteria;p__Bacteroidota;c__Bacteroidia;o__Flavobacteriales;f__Flavobacteriaceae;g__Flavobacterium;s__	93.2	0.8	3.09	264	11,718	34	2	40	2	0	0	0.04808	0.01753	0.05116	0.03892	SAMN18059850	JAFLBT000000000	PRJNA704939
UWPOB_FLAVO2	GCA_017303755.1	FLAVO2	d__Bacteria;p__Bacteroidota;c__Bacteroidia;o__Flavobacteriales;f__Flavobacteriaceae;g__Flavobacterium;s__	100	0	4.07	15	271,271	34.7	1	46	1	0	0	0.44824	0.18295	0.09632	0.2425	SAMN18059849	JAFLBS000000000	PRJNA704939
UWPOB_FLAVO3	GCA_017303695.1	FLAVO3	d__Bacteria;p__Bacteroidota;c__Bacteroidia;o__Flavobacteriales;f__Weeksellaceae;g__Chryseobacterium_A;s__Chryseobacterium_A	100	0	2.57	12	214,243	36.8	3	37	2	0	1	0.38721	0.17728	0.00755	0.19068	SAMN18059851	JAFLBU000000000	PRJNA704939
UWPOB_IGNAVI1	GCA_017302895.1	IGNAVI1	d__Bacteria;p__Bacteroidota;c__Ignavibacteria;o__Ignavibacteriales;f__Ignavibacteriaceae_A;g__UTCHB3;s__	100	0	3.98	6	663,902	42.2	3	44	1	1	1	2.23238	0.35053	0.08219	0.88837	SAMN18059852	JAFLBV000000000	PRJNA704939
UWPOB_IGNAVI2	GCA_017302885.1	IGNAVI2	d__Bacteria;p__Bacteroidota;c__Ignavibacteria;o__SJA-28;f__;g__;s__	95.8	2.1	4.73	460	10,272	35.6	4	51	2	1	1	0.0987	0.13661	0.18405	0.13979	SAMN18059853	JAFLBW000000000	PRJNA704939
UWPOB_IGNAVI3	GCA_017303675.1	IGNAVI3	d__Bacteria;p__Bacteroidota;c__Ignavibacteria;o__SJA-28;f__OLB5;g__OLB5;s__	93.1	0.6	3.65	3	1,217,215	37.8	3	43	1	1	1	13.6012	18.6822	18.7649	17.0161	SAMN18059854	JAFLBX000000000	PRJNA704939
UWPOB_KAPA1	GCA_017302855.1	KAPA1	d__Bacteria;p__Bacteroidota;c__Kapabacteria;o__Kapabacteriales;f__UBA961;g__UBA2353;s__	93.5	0	3.6	309	11,645	38.6	0	34	0	0	0	0.0477	0.02835	0.0537	0.04325	SAMN18059855	JAFLBY000000000	PRJNA704939
UWPOB_RHODO1	GCA_017303715.1	RHODO1	d__Bacteria;p__Bacteroidota;c__Rhodothermia;o__Rhodothermales;f__UBA2364;g__UBA2364;s__	100	0	4.35	62	70,212	46.1	3	42	1	1	1	1.30416	0.50553	1.2655	1.02506	SAMN18059856	JAFLBZ000000000	PRJNA704939
UWPOB_DELTA1	GCA_017302875.1	DELTA1	d__Bacteria;p__Bdellovibrionota_B;c__UBA2361;o__UBA2361;f__;g__;s__	95.7	3.8	3.86	296	13,034	52.6	3	45	1	1	1	0.16581	0.1972	0.0576	0.14021	SAMN18059857	JAFLCA000000000	PRJNA704939
UWPOB_DELTA2	GCA_017302795.1	DELTA2	d__Bacteria;p__Bdellovibrionota;c__Bdellovibrionia;o__Bdellovibrionales;f__Bdellovibrionaceae;g__Ga0074137;s__	100	0	4.23	39	108,584	49.2	3	39	1	1	1	0.02329	0.29036	2.53023	0.94796	SAMN18059858	JAFLCB000000000	PRJNA704939
UWPOB_DELTA3	GCA_017302835.1	DELTA3	d__Bacteria;p__Bdellovibrionota;c__Bdellovibrionia;o__Bdellovibrionales;f__Bdellovibrionaceae;g__UBA2316;s__	99.1	0.9	3.63	35	103,678	37.3	3	32	1	1	1	0.73	0.4205	0.04244	0.39765	SAMN18059859	JAFLCC000000000	PRJNA704939
UWPOB_DELTA4	GCA_017302715.1	DELTA4	d__Bacteria;p__Bdellovibrionota;c__UBA2394;o__UBA2394;f__UBA2394;g__;s__	100	0	2.39	21	113,698	41.4	4	41	1	1	2	0.09683	0.07426	0.01366	0.06158	SAMN18059860	JAFLCD000000000	PRJNA704939
UWPOB_ANAER1	GCA_017303655.1	ANAER1	d__Bacteria;p__Chloroflexota;c__Anaerolineae;o__Anaerolineales;f__envOPS12;g__OLB14;s__	83.1	6	4.61	172	26,782	49.6	1	40	1	0	0	0.78326	0.58803	0.33592	0.56907	SAMN18059861	JAFLCE000000000	PRJNA704939
UWPOB_ANAER2	GCA_017303735.1	ANAER2	d__Bacteria;p__Chloroflexota;c__Anaerolineae;o__Anaerolineales;f__envOPS12;g__OLB14;s__	87.5	6.4	4.8	421	11,388	50	0	47	0	0	0	0.46474	0.39027	0.53912	0.46471	SAMN18059862	JAFLCF000000000	PRJNA704939
UWPOB_ANAER3	GCA_017303775.1	ANAER3	d__Bacteria;p__Chloroflexota;c__Anaerolineae;o__SBR1031;f__A4b;g__;s__	85.2	0	4.47	499	8,952	56	2	29	2	0	0	0.22893	0.18326	0.09188	0.16802	SAMN18059864	JAFLCH000000000	PRJNA704939
UWPOB_ANAER4	GCA_017303635.1	ANAER4	d__Bacteria;p__Chloroflexota;c__Anaerolineae;o__SBR1031;f__A4b;g__;s__	99.5	0	6.8	39	174,361	56.3	3	48	1	1	1	0.50118	0.47335	0.26963	0.41472	SAMN18059863	JAFLCG000000000	PRJNA704939
UWPOB_ANAER5	GCA_017304075.1	ANAER5	d__Bacteria;p__Chloroflexota;c__Anaerolineae;o__SBR1031;f__A4b;g__OLB15;s__	91.7	5.5	7.11	706	10,069	60.1	2	45	2	0	0	0.16356	0.17579	0.09332	0.14422	SAMN18059865	JAFLCI000000000	PRJNA704939
UWPOB_LEPTO1	GCA_017302695.1	LEPTO1	d__Bacteria;p__Cyanobacteria;c__Cyanobacteriia;o__Leptolyngbyales;f__Leptolyngbyaceae;g__Leptolyngbya;s__Leptolyngbya	100	0	6.8	61	111,481	46.9	0	54	0	0	0	0.57222	0.82313	0.90187	0.76574	SAMN18059866	JAFLCJ000000000	PRJNA704939
UWPOB_OBS1	GCA_017303615.1	OBS1	d__Bacteria;p__Cyanobacteria;c__Vampirovibrionia;o__Obscuribacterales;f__Obscuribacteraceae;g__Obscuribacter;s__Obscuribacter	96.6	0.9	5.06	70	72,324	49	3	42	1	1	1	0.22519	0.60208	1.10521	0.64416	SAMN18059867	JAFLCK000000000	PRJNA704939
UWPOB_DELTA5	GCA_017303575.1	DELTA5	d__Bacteria;p__Myxococcota;c__Polyangia;o__Polyangiales;f__Sandaracinaceae;g__;s__	95.8	0	9.39	540	17,383	70.1	3	71	1	1	1	0.04541	0.10081	0.34331	0.16318	SAMN18059868	JAFLCL000000000	PRJNA704939
UWPOB_PLANC1	GCA_017303565.1	PLANC1	d__Bacteria;p__Planctomycetota;c__Phycisphaerae;o__Phycisphaerales;f__SM1A02;g__;s__	88.3	6.3	2.93	530	5,521	68.6	1	37	0	1	0	0.02712	0.0326	0.0993	0.053	SAMN18059869	JAFLCM000000000	PRJNA704939
UWPOB_PLANC2	GCA_017303555.1	PLANC2	d__Bacteria;p__Planctomycetota;c__Phycisphaerae;o__Phycisphaerales;f__SM1A02;g__UBA2402;s__	95.8	0	4.06	42	96,645	63.5	3	48	1	1	1	0.35589	0.44712	0.11479	0.30593	SAMN18059870	JAFLCN000000000	PRJNA704939
UWPOB_PLAC3	GCA_017303535.1	PLANC3	d__Bacteria;p__Planctomycetota;c__Planctomycetes;o__Pirellulales;f__;g__;s__	93.5	1.3	6.61	641	10,313	61.8	3	71	1	1	1	0.02337	0.03888	0.08353	0.0486	SAMN18059871	JAFLCO000000000	PRJNA704939
UWPOB_PLANC4	GCA_017303505.1	PLANC4	d__Bacteria;p__Planctomycetota;c__Planctomycetes;o__Pirellulales;f__Pirellulaceae;g__Pirellula_B;s__	96.4	0.2	7.83	308	25,424	50.3	0	98	0	0	0	0.02843	0.10226	0.04436	0.05835	SAMN18059872	JAFLCP000000000	PRJNA704939
UWPOB_PLANC5	GCA_017302815.1	PLANC5	d__Bacteria;p__Planctomycetota;c__UBA11346;o__UBA11346;f__UBA11346;g__UBA11346;s__	78	2	4.28	568	7,540	69.7	0	74	0	0	0	0.14524	0.27428	0.04468	0.15473	SAMN18059873	JAFLCQ000000000	PRJNA704939
UWPOB_ALPHA1	GCA_017302755.1	ALPHA1	d__Bacteria;p__Proteobacteria;c__Alphaproteobacteria;o__;f__;g__;s__	94.8	0	2.12	275	7,722	59.4	2	35	1	0	1	0.00365	0.0307	0.09852	0.04429	SAMN18059874	JAFLCR000000000	PRJNA704939
UWPOB_CAULO1	GCA_017302335.1	CAULO1	d__Bacteria;p__Proteobacteria;c__Alphaproteobacteria;o__Caulobacterales;f__Caulobacteraceae;g__Brevundimonas;s__	96.6	0.7	2.6	168	15,478	67.9	1	44	0	1	0	0.29751	0.15141	0.02773	0.15888	SAMN18059876	JAFLCT000000000	PRJNA704939
UWPOB_CAULO2	GCA_017302105.1	CAULO2	d__Bacteria;p__Proteobacteria;c__Alphaproteobacteria;o__Caulobacterales;f__Caulobacteraceae;g__Brevundimonas;s__	100	0	2.57	12	214,253	65.7	3	38	1	1	1	1.58091	0.90968	0.11758	0.86939	SAMN18059875	JAFLCS000000000	PRJNA704939
UWPOB_CAULO3	GCA_017303355.1	CAULO3	d__Bacteria;p__Proteobacteria;c__Alphaproteobacteria;o__Caulobacterales;f__Hyphomonadaceae;g__SWB02;s__	98.4	0.7	3.67	34	107,997	63.9	3	47	1	1	1	0.9416	0.71847	0.41979	0.69328	SAMN18059877	JAFLCU000000000	PRJNA704939
UWPOB_CAULO4	GCA_017303435.1	CAULO4	d__Bacteria;p__Proteobacteria;c__Alphaproteobacteria;o__Caulobacterales;f__Hyphomonadaceae;g__UBA1942;s__	75	6.3	3.49	362	9,650	39.2	0	35	0	0	0	0.09806	0.03402	0.00279	0.04496	SAMN18059878	JAFLCV000000000	PRJNA704939
UWPOB_MICAV1	GCA_017302165.1	MICAV1	d__Bacteria;p__Proteobacteria;c__Alphaproteobacteria;o__Micavibrionales;f__Micavibrionaceae;g__;s__	96	0.2	2	6	333,677	49.3	2	40	0	1	1	0.02039	0.01146	0.35063	0.1275	SAMN18059879	JAFLCW000000000	PRJNA704939
UWPOB_RHIZO1	GCA_017302635.1	RHIZO1	d__Bacteria;p__Proteobacteria;c__Alphaproteobacteria;o__Rhizobiales;f__Beijerinkiaceae_A;g__PAR1;s__	97.3	0.6	3.1	6	516,587	62.1	3	47	1	1	1	0.21701	0.24399	0.35215	0.27105	SAMN18059880	JAFLCX000000000	PRJNA704939
UWPOB_RHODOB1	GCA_017303485.1	RHODOB1	d__Bacteria;p__Proteobacteria;c__Alphaproteobacteria;o__Rhodobacterales;f__Rhodobacteraceae;g__Tabrizicola;s__	96.6	8.6	4.39	272	16,121	65.3	0	53	0	0	0	0.06267	0.10359	0.03424	0.06683	SAMN18059881	JAFLCY000000000	PRJNA704939
UWPOB_RICK1	GCA_017302665.1	RICK1	d__Bacteria;p__Proteobacteria;c__Alphaproteobacteria;o__Rickettsiales;f__Rickettsiaceae;g__;s__	98.6	7.1	1.65	29	56,773	33.3	4	34	1	1	2	0.00855	0.16939	0.03581	0.07125	SAMN18059882	JAFLCZ000000000	PRJNA704939
UWPOB_RICK2	GCA_017302595.1	RICK2	d__Bacteria;p__Proteobacteria;c__Alphaproteobacteria;o__Rickettsiales;f__Rickettsiaceae;g__GCA-2402195;s__	99.5	1	1.33	5	264,993	34.7	0	34	0	0	0	0.03107	0.20695	0.08658	0.1082	SAMN18059883	JAFLDA000000000	PRJNA704939
UWPOB_SPHING1	GCA_017302775.1	SPHING1	d__Bacteria;p__Proteobacteria;c__Alphaproteobacteria;o__Sphingomonadales;f__Sphingomonadaceae;g__Novosphingobium;s__	78.5	5.1	3.16	556	5,684	61.6	0	48	0	0	0	0.06166	0.0599	0.01391	0.04516	SAMN18059885	JAFLDC000000000	PRJNA704939
UWPOB_SPHING2	GCA_017302615.1	SPHING2	d__Bacteria;p__Proteobacteria;c__Alphaproteobacteria;o__Sphingomonadales;f__Sphingomonadaceae;g__Novosphingobium;s__	99.2	0.1	3.02	33	91,486	65.7	3	46	1	1	1	0.6247	0.62665	0.23261	0.49465	SAMN18059884	JAFLDB000000000	PRJNA704939
UWPOB_ALPHA2	GCA_017302575.1	ALPHA2	d__Bacteria;p__Proteobacteria;c__Alphaproteobacteria;o__UBA998;f__UBA3002;g__;s__	98.9	0	1.86	1	1,861,815	50.8	3	39	1	1	1	0.47594	0.19689	0.04249	0.23844	SAMN18059886	JAFLDD000000000	PRJNA704939
UWPOB_BURK1	GCA_017302655.1	BURK1	d__Bacteria;p__Proteobacteria;c__Gammaproteobacteria;o__Burkholderiales;f__Burkholderiaceae;g__;s__	100	0	4.44	203	21,857	67.3	2	48	1	1	0	0.37852	0.42244	0.55223	0.45106	SAMN18059887	JAFLDE000000000	PRJNA704939
UWPOB_OTTO1	GCA_017302725.1	OTTO1	d__Bacteria;p__Proteobacteria;c__Gammaproteobacteria;o__Burkholderiales;f__Burkholderiaceae;g__Ottowia;s__Ottowia	95.8	0	2.87	325	8,835	68.6	3	52	1	1	1	0.76562	0.83948	0.30961	0.63823	SAMN18059888	JAFLDF000000000	PRJNA704939
UWPOB_RUBRI1	GCA_017302495.1	RUBRI1	d__Bacteria;p__Proteobacteria;c__Gammaproteobacteria;o__Burkholderiales;f__Burkholderiaceae;g__Rubrivivax;s__	81	0.8	4.51	553	8,152	71.1	3	56	1	1	1	1.62184	1.49067	0.45613	1.18955	SAMN18059889	JAFLDG000000000	PRJNA704939
UWPOB_RUBRI2	GCA_017302505.1	RUBRI2	d__Bacteria;p__Proteobacteria;c__Gammaproteobacteria;o__Burkholderiales;f__Burkholderiaceae;g__Rubrivivax;s__	100	0	5.5	555	9,913	68.4	4	52	2	1	1	0.21114	0.24105	0.10683	0.18634	SAMN18059890	JAFLDH000000000	PRJNA704939
UWPOB_VITRI1	GCA_017302485.1	VITRI1	d__Bacteria;p__Proteobacteria;c__Gammaproteobacteria;o__Burkholderiales;f__Burkholderiaceae;g__Vitreoscilla_A;s__	95.8	0	3.68	513	7,177	68.4	1	42	1	0	0	0.07383	0.05699	0.17722	0.10268	SAMN18059891	JAFLDI000000000	PRJNA704939
UWPOB_BURK2	GCA_017302475.1	BURK2	d__Bacteria;p__Proteobacteria;c__Gammaproteobacteria;o__Burkholderiales;f__Palsa-1005;g__;s__	79.2	0	2.71	498	5,448	69.6	0	34	0	0	0	0.10158	0.12158	0.11145	0.11154	SAMN18059892	JAFLDJ000000000	PRJNA704939
UW10-POB	GCA_017302555.1	CAPIIB	d__Bacteria;p__Proteobacteria;c__Gammaproteobacteria;o__Burkholderiales;f__Rhodocyclaceae;g__Accumulibacter;s__	100	0	4.4	88	49,949	62.5	0	41	0	0	0	0.52449	0.60697	0.65232	0.59459	SAMN18059893	JAFLDK000000000	PRJNA704939
UW12-POB	GCA_017302435.1	CAPIID	d__Bacteria;p__Proteobacteria;c__Gammaproteobacteria;o__Burkholderiales;f__Rhodocyclaceae;g__Accumulibacter;s__	98.1	0.7	4.56	91	50,155	62.7	0	46	0	0	0	0.35414	0.32156	0.05167	0.24246	SAMN18059894	JAFLDL000000000	PRJNA704939
UW13-POB	GCA_017302415.1	CAPIIF	d__Bacteria;p__Proteobacteria;c__Gammaproteobacteria;o__Burkholderiales;f__Rhodocyclaceae;g__Accumulibacter;s__	97.1	4.4	5.45	245	22,232	65.9	1	48	0	1	0	1.65004	0.70231	0.09466	0.81567	SAMN18059895	JAFLDM000000000	PRJNA704939
UW11-POB	GCA_017302385.1	CAPIIC	d__Bacteria;p__Proteobacteria;c__Gammaproteobacteria;o__Burkholderiales;f__Rhodocyclaceae;g__Accumulibacter;s__Accumulibacter	100	0	4.63	144	32,127	61.2	0	47	0	0	0	12.2117	11.8669	10.2307	11.4364	SAMN18059898	JAFLDP000000000	PRJNA704939
UW8-POB	GCA_017302345.1	CAPIA	d__Bacteria;p__Proteobacteria;c__Gammaproteobacteria;o__Burkholderiales;f__Rhodocyclaceae;g__Accumulibacter;s__Accumulibacter	100	0	4.26	49	86,993	64.2	0	51	0	0	0	6.34328	6.19256	0.4336	4.32315	SAMN18059896	JAFLDN000000000	PRJNA704939
UW9-POB	GCA_017302455.1	CAPIIA	d__Bacteria;p__Proteobacteria;c__Gammaproteobacteria;o__Burkholderiales;f__Rhodocyclaceae;g__Accumulibacter;s__Accumulibacter	100	0	4.93	126	39,138	64.1	1	46	1	0	0	0.55782	0.54654	0.15933	0.42123	SAMN18059897	JAFLDO000000000	PRJNA704939
UWPOB_DECH1	GCA_017302355.1	DECH1	d__Bacteria;p__Proteobacteria;c__Gammaproteobacteria;o__Burkholderiales;f__Rhodocyclaceae;g__Dechloromonas;s__	91.5	8.1	3.69	279	13,228	65	2	40	0	1	1	2.09698	3.33708	3.17482	2.86963	SAMN18059899	JAFLDQ000000000	PRJNA704939
UWPOB_SULF1	GCA_017302275.1	SULF1	d__Bacteria;p__Proteobacteria;c__Gammaproteobacteria;o__Burkholderiales;f__Rhodocyclaceae;g__Sulfuritalea;s__	98.3	1.1	4.41	88	50,106	64	3	47	2	1	0	0.76164	0.92706	2.2433	1.31067	SAMN18059900	JAFLDR000000000	PRJNA704939
UWPOB_THAU1	GCA_017302295.1	THAU1	d__Bacteria;p__Proteobacteria;c__Gammaproteobacteria;o__Burkholderiales;f__Rhodocyclaceae;g__Thauera;s__	98.7	1.4	4.11	56	73,417	66.1	3	52	3	0	0	0.5635	0.57917	0.40828	0.51699	SAMN18059901	JAFLDS000000000	PRJNA704939
UWPOB_ZOOG1	GCA_017309145.1	ZOOG1	d__Bacteria;p__Proteobacteria;c__Gammaproteobacteria;o__Burkholderiales;f__Rhodocyclaceae;g__Zoogloea;s__	83.3	4.2	4.91	428	11,461	65.7	0	58	0	0	0	0.06958	0.06569	0.07688	0.07071	SAMN18059903	JAFLDU000000000	PRJNA704939
UWPOB_ZOOG2	GCA_017302315.1	ZOOG2	d__Bacteria;p__Proteobacteria;c__Gammaproteobacteria;o__Burkholderiales;f__Rhodocyclaceae;g__Zoogloea;s__	95.2	1.8	4.52	165	27,407	64.5	0	55	0	0	0	0.12938	0.14704	0.21305	0.16316	SAMN18059902	JAFLDT000000000	PRJNA704939
UWPOB_PARA1	GCA_017302255.1	PARA1	d__Bacteria;p__Proteobacteria;c__Gammaproteobacteria;o__Enterobacterales;f__Alteromonadaceae;g__Pararheinheimera;s__	100	4.2	4.03	77	52,358	46.2	0	50	0	0	0	0.20334	0.02657	0.00492	0.07828	SAMN18059904	JAFLDV000000000	PRJNA704939
UWPOB_GAMMA1	GCA_017302205.1	GAMMA1	d__Bacteria;p__Proteobacteria;c__Gammaproteobacteria;o__GCA-2729495;f__GCA-2729495;g__;s__	79.2	1	2.51	456	5,508	69.2	1	34	1	0	0	0.05103	0.07089	0.10094	0.07429	SAMN18059905	JAFLDW000000000	PRJNA704939
UWPOB_RHODAN1	GCA_017302215.1	RHODAN1	d__Bacteria;p__Proteobacteria;c__Gammaproteobacteria;o__Xanthomonadales;f__Rhodanobacteraceae;g__Dokdonella;s__	91.7	0	3.5	323	10,843	68.8	0	49	0	0	0	0.144	0.20595	0.16775	0.17257	SAMN18059906	JAFLDX000000000	PRJNA704939
UWPOB_AQUI1	GCA_017302155.1	AQUI1	d__Bacteria;p__Proteobacteria;c__Gammaproteobacteria;o__Xanthomonadales;f__Xanthomonadaceae;g__Aquimonas;s__Aquimonas	98.3	1.6	3.98	113	35,212	61.5	0	46	0	0	0	0.28393	0.24393	0.06593	0.19793	SAMN18059907	JAFLDY000000000	PRJNA704939
UWPOB_LYSO1	GCA_017302135.1	LYSO1	d__Bacteria;p__Proteobacteria;c__Gammaproteobacteria;o__Xanthomonadales;f__Xanthomonadaceae;g__Lysobacter_A;s__	99.6	1	4.34	35	123,959	64.1	2	48	1	1	0	1.21091	1.20058	0.96205	1.12451	SAMN18059908	JAFLDZ000000000	PRJNA704939
UWPOB_PSEUDO1	GCA_017302035.1	PSEUDO1	d__Bacteria;p__Proteobacteria;c__Gammaproteobacteria;o__Xanthomonadales;f__Xanthomonadaceae;g__Pseudoxanthomonas;s__	95.8	0	2.91	125	23,300	69.3	1	48	1	0	0	1.50336	2.17593	0.47299	1.38409	SAMN18059909	JAFLEA000000000	PRJNA704939
UWPOB_THERMO1	GCA_017302095.1	THERMO1	d__Bacteria;p__Proteobacteria;c__Gammaproteobacteria;o__Xanthomonadales;f__Xanthomonadaceae;g__Thermomonas;s__	76.4	2.1	2.31	299	7,734	69.7	0	39	0	0	0	0.21965	0.25626	0.05906	0.17832	SAMN18059911	JAFLEC000000000	PRJNA704939
UWPOB_THERMO2	GCA_017302075.1	THERMO2	d__Bacteria;p__Proteobacteria;c__Gammaproteobacteria;o__Xanthomonadales;f__Xanthomonadaceae;g__Thermomonas;s__	87.5	0.5	2.77	245	11,303	69.9	0	41	0	0	0	0.79368	1.14049	0.4586	0.79759	SAMN18059910	JAFLEB000000000	PRJNA704939
UWPOB_SPIRO1	GCA_017302045.1	SPIRO1	d__Bacteria;p__Spirochaetota;c__Leptospirae;o__Turneriellales;f__Turneriellaceae;g__Turneriella;s__	86.5	0	3.78	412	9,184	53.8	2	31	2	0	0	0.03758	0.03209	0.0621	0.04392	SAMN18059912	JAFLED000000000	PRJNA704939
UWPOB_SPIRO2	GCA_017302015.1	SPIRO2	d__Bacteria;p__Spirochaetota;c__UBA12135;o__;f__;g__;s__	87.5	0	5.43	173	31,368	65.2	2	41	0	1	1	0.09083	0.09013	0.07872	0.08656	SAMN18059913	JAFLEE000000000	PRJNA704939
UWPOB_VERRUCO1	GCA_017302195.1	VERRUCO1	d__Bacteria;p__Verrucomicrobiota;c__Verrucomicrobiae;o__Pedosphaerales;f__UBA9464;g__UBA9464;s__	81	8.3	4.86	848	5,726	67.1	0	35	0	0	0	0.04712	0.07195	0.01614	0.04507	SAMN18059915	JAFLEG000000000	PRJNA704939
UWPOB_VERRUCO2	GCA_017309115.1	VERRUCO2	d__Bacteria;p__Verrucomicrobiota;c__Verrucomicrobiae;o__Pedosphaerales;f__UBA9464;g__UBA9464;s__	98.6	3.5	7.53	112	67,228	61.5	3	50	1	1	1	0.14886	0.18964	0.1474	0.16196	SAMN18059914	JAFLEF000000000	PRJNA704939
UWPOB_VERRUCO3	GCA_017301995.1	VERRUCO3	d__Bacteria;p__Verrucomicrobiota;c__Verrucomicrobiae;o__Verrucomicrobiales;f__Akkermansiaceae;g__UBA1315;s__	100	0	7.04	68	103,585	62.3	2	60	2	0	0	0.30515	0.26629	0.07792	0.21646	SAMN18059916	JAFLEH000000000	PRJNA704939
UWPOB_VERRUCO4	GCA_017301975.1	VERRUCO4	d__Bacteria;p__Verrucomicrobiota;c__Verrucomicrobiae;o__Verrucomicrobiales;f__Verrucomicrobiaceae;g__Prosthecobacter;s__	97	0	6.9	97	71,172	60.3	0	54	0	0	0	0.17845	0.15859	0.07204	0.13636	SAMN18059917	JAFLEI000000000	PRJNA704939

a Genome information for 86 metagenome-assembled genomes is shown. The column labeled “Isolate name” lists the name submitted to NCBI, and that labeled “Code” lists the codes used in Fig. 1. Classifications were assigned with GTDB-tk and “*Candidatus* Accumulibacter phosphatis” clade designations based on comparisons to *ppk1* clones and publicly available reference genome sequences. “*Candidatus* Accumulibacter phosphatis” genomes are named alphanumerically according to previous nomenclature; all other genomes are named UWPOB_CODE. The genome statistics for completeness, contamination, GC content, genome size, and number of contigs were performed with CheckM v1.1.2. The rRNA and tRNA genes were predicted with Barrnap v0.9, as part of the Prokka v1.13.7 package. The relative abundance was calculated using coverM v0.4.0 using the relative_abundance method for the three metagenomic samples, taken on 16 July 2015, 24 July 2015, and 6 August 2015 toward the end of the enrichment period, and averaged together across all three samples for an average relative abundance calculation.

### Data availability.

The raw metagenomes for the 3 samples and genome assemblies for all 86 metagenome-assembled genomes (MAGs) are available at NCBI GenBank under BioProject accession number PRJNA704939. The metagenomes are available under the SRA accession numbers SRR13786854, SRR13786855, and SRR13786856.
